# Off-label prescribing in ophthalmology: a retrospective analysis

**DOI:** 10.3389/fphar.2026.1837220

**Published:** 2026-06-11

**Authors:** Claudio Bucolo, Lucia Gozzo, Laura Terranova, Maria Rita Badagliacca, Giovanni Luca Romano, Alessandro Avitabile, Andrea Russo, Antonio Lazzara, Filippo Drago, Federica Conti

**Affiliations:** 1 Department of Biomedical and Biotechnological Sciences, University of Catania, Catania, Italy; 2 Pharmacy Operative Complex Unit, University Hospital “G. Rodolico”, Catania, Italy; 3 Centre for Research and Consultancy in HTA and Drug Regulatory Affairs (CERD), University of Catania, Catania, Italy; 4 HTA Commission, University Hospital of Catania, Catania, Italy; 5 Pharmacy Operative Complex Unit, University Hospital “San Marco”, Catania, Italy; 6 Department of Medicine and Surgery, University of Enna “Kore”, Enna, Italy; 7 Department of Ophthalmology, University of Palermo, Palermo, Italy; 8 Department of Ophthalmology, University of Catania, Catania, Italy; 9 Health Department, University Hospital of Catania, Catania, Italy

**Keywords:** anti-vascular endothelial growth factor (VEGF), eye, ocular diseases, off-label drugs, ophthalmology

## Abstract

Off-label practice is often needed in ophthalmology, due to the limited number of drugs approved to treat several ocular diseases and to manage challenging ocular surgeries. The aim was to analyze the off-label drug prescriptions issued from January 2014 to December 2024 at the Department of Ophthalmology of the University Hospital of Catania (Italy). 233 off-label prescriptions, comprising 13 drugs, were delivered to treat 225 patients affected by ocular disorders. Results of the present study showed a raise of off-label prescriptions in 2015 and 2016 mainly due to mitomycin C use in trabeculectomy, representing 34.3% of the total off-label drug prescriptions in the reference period. After a decrease in 2017–2018, there was an increasing trend in the number of off-label issued prescriptions until 2024. The main contributors were anti-vascular endothelial growth factor (VEGF) drugs such as bevacizumab, ranibizumab and aflibercept, which represent 25.3%, 9.0% and 4.3% of off-label prescriptions, respectively. Additionally, 5-fluorouracil and dexamethasone were prescribed off-label, each representing 7.3% of the total off-label prescriptions, to mainly handle conjunctival carcinoma and various macular edema, respectively. Alteplase (6.9%), voriconazole (2.1%), verteporfin (1.3%), and foscarnet (0.9%), together with amphotericin B (0.4%), ganciclovir (0.4%), as well as rituximab (0.4%) were off-label prescribed to manage other specific ocular conditions. These results suggest that off-label practice is evolving in the field of ophthalmology. Collaboration among regulatory authorities, clinicians, marketing authorization holders, and patients is essential, in order to develop standardized, evidence-based international guidelines about off-label use, to improve outcome of patients with unmet medical needs.

## 1 Introduction

Off-label drug use is referred to the usage of medications that fall outside the approved indications, dosages, routes of administration, treatments durations, or patients’ populations (Vrancken, 2015). The aim of off-label prescribing is to ensure the utmost benefit to the patients, especially for diseases with no treatment available or when approved therapies have failed. Indeed, one of the main advantages of off-label practice is that it increases the access to medications for special patients’ categories, satisfying the unmet medical needs by the conventional therapies (Rusz et al., 2021). Considering the long-development times and high costs to obtain drug approval, off-label prescribing is a recognized worldwide practice. Noteworthy, off-label drugs prescription is widely employed in different clinical areas, as well as rare diseases, psychiatry, oncology, pediatrics, and even ophthalmology (Saiyed et al., 2017; Fung et al., 2021; Rusz et al., 2021; Hefner et al., 2022; Li et al., 2022; Meng et al., 2022). This latter is often neglected because is a niche field with few numbers of available drugs (Bucolo and Schmetterer, 2016).

Currently, there are no uniform international guidelines or regulations for off-label drug use, but some countries adopted specific laws to guarantee patients’ wellbeing and reduce unmotivated risks related to off-label prescriptions (Marjolein, 2017; Gozzo et al., 2020). Indeed, although off-label use allows to satisfy unmet medical needs, it could increase the risk of inappropriate use and medical error due to the lack of a defined risk-benefit ratio (Bellis et al., 2014). Therefore, appropriateness of these prescriptions must be carefully assessed to ensure that such use occurs only if supported by data demonstrating a favorable risk/benefit profile.

In Italy, a comprehensive body of legislation has been introduced to regulate off-label use. The Law 94/1998 allows physician, under his/her direct responsibility, to prescribe a drug not in accordance with the Summary of Product Characteristics (SmPC) in individual and exceptional cases based on efficacy and safety data (Law 94, 1998). The Law establishes that patients must be adequately informed and need to pay the cost of treatment, not covered by national health system (NHS), excluding those treated in the hospital setting, where costs are covered within the hospital budget.

On the contrary, the Law 648/1996 provides the list of off-label drugs reimbursed by the Italian NHS, based on new scientific evidence resulting from at least phase II clinical trials (Law 648, 1996).

In compliance with the national laws, the clinical pharmacologists ensure the appropriateness of off-label drug prescriptions performed by clinicians at the University Hospital of Catania (Italy). The aim of this study is to provide a description of off-label drug use in ophthalmology according to Law 94/1998 at the University Hospital of Catania.

## 2 Methods

A retrospective analysis of administrative data collected from January 2014 to December 2024 was conducted to identify off-label drugs prescribed in ophthalmology according to Law 94/1998, at the Department of Ophthalmology of Catania University Hospital, including both inpatients and outpatients.

The drugs were considered as “off-label” if used for a therapeutic indication different from the approved one, as well as for different dosage, dosing frequency or duration of use, method of administration and patient group (e.g., children instead of adults). The data collected included age, sex, therapeutic indication, prescribed drugs, dosage form, frequency of administration, number of treated eyes, administration route.

The data collection and analysis were carried out for administrative purposes, in order to control and monitor off-label use in the hospital setting. For this reason, according to the guideline for the classification and conduct of observational studies issued by the Italian Medicines Agency (AIFA), ethics committee approval was not required (AIFA, 2024). Additionally, Summary of Product Characteristics (SmPCs) were obtained from EMA website referring to the year of drug prescription.

Descriptive statistic was performed, reporting frequencies and percentages to summarize categorical data and mean and median values for continuous data.

Microsoft Excel 2021 and GraphPad prism nine software were used to collect and analyze data.

## 3 Results

In the reference period, a total of 233 off-label prescriptions (comprising 13 drugs) were issued for 225 patients at the Department of Ophthalmology of the University Hospital of Catania. Among them, 97 were female (43.1%) and 128 were male (56.9%). The average age was 57.9 years (median age 62.5, range 0–91). The number of eyes treated were 243.

**FIGURE 1:**
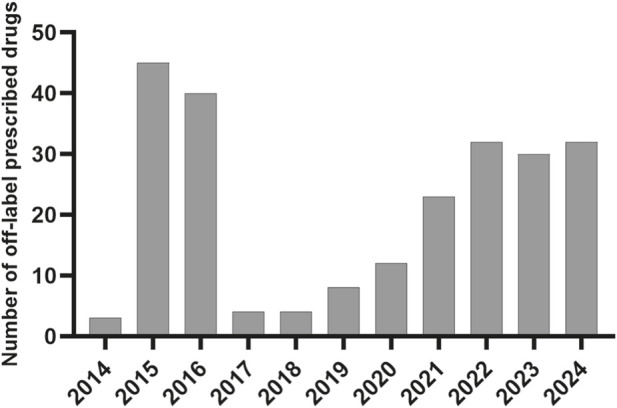
Off-label prescriptions in ophthalmology from January 2014 to December 2024. Off-label prescribed drugs in ophthalmology were higher particularly in 2015 and 2016 years. Subsequently to 2017 and 2018 years, there was an increasing trend in off-label ophthalmic prescriptions, then the prescriptions were nearly constant in the last 3 years (2022–2024).

The number of prescriptions showed an increase in 2015 (n = 45, 19.3%) and 2016 (n = 40, 17.2%; Figure 1). Subsequently, the number of off-label issued was nearly constant in 2022, 2023 and 2024, with 32 (13.7%), 30 (12.9%) and 32 (13.7%) prescriptions, respectively. Anti-vascular endothelial growth factor (VEGF) drugs were the most prescribed off-label intravitreal treatment (n = 90, 38.6%; Table 1) mainly for patients with different types of macular edema not included in the SmPCs, and with neovascular glaucoma (Tables 2, 3). Mitomycin C was one of the most prescribed drugs (n = 80, 34.3%, Table 1), extensively used as antifibrotic coadjutant in trabeculectomy through topical intraoperative application (Table 3). 5-fluorouracil, employed as eye drops for ocular squamous cell carcinoma (Table 2), and intravitreal dexamethasone implant, administered for macular edema outside the posology approved in the SmPC (Table 3), represent the 7.3% of the total off-label drug prescriptions (both n = 17 prescriptions, Table 1). 16 prescriptions have been recorded for alteplase (6.9%, Table 1) to treat subretinal macular hemorrhage with intravitreal injections (Table 2). Voriconazole (n = 5, 2.1%, Table 1), both as eye drops and intravitreal injections, and amphotericin B (n = 1, 0.4%, Table 1), as eye drops, have been prescribed for ocular *candida* infections (Tables 3, 2). Whereas intravitreal foscarnet (n = 2, 0.9%, Table 1) and ganciclovir (n = 1, 0.4%, Table 1) have been employed for viral infections (Table 3). Lastly, three patients with central serous chorioretinopathy (Table 3) were intravenous treated with verteporfin (1.3%, Table 1), and 1 with ocular pemphigoid (Tables 1, 3) was treated with rituximab through intravenous injection.

**TABLE 1:** Frequency, percentage of off-label prescriptions in ophthalmology in 11 years (2014–2024) and respectively drug administration routes.

Drug	N	%	Administration routes
Anti-VEGF drugs	90	38,6	Intravitreal
Bevacizumab	59	25.3	
Ranibizumab	21	9	
Aflibercept	10	4.3	
Mitomycin C	80	34.3	Topical intraoperative application
5-Fluorouracil	17	7.3	Eye drops
Dexamethasone	17	7.3	Intravitreal implant
Alteplase	16	6.9	Intravitreal
Voriconazole	5	2.1	Eye drops/intravitreal
Verteporfin	3	1.3	Intravenous
Foscarnet	2	0.9	Intravitreal
Amphotericin B	1	0.4	Eye drops
Ganciclovir	1	0.4	Intravitreal
Rituximab	1	0.4	Intravenous
Total	233	100	​

**TABLE 2:** Off-label drugs prescribed in specific ocular conditions (part I).

Drug	Pathology
5-fluorouracil	Conjunctivocorneal squamous cell carcinoma
	Squamous cell carcinoma of the conjunctiva
Aflibercept	Cystoid macular edema in irvine-gass syndrome
	Inflammatory macular edema
	Macular edema associated with hamartoma
	Macular edema secondary to pneumatic retinopexy for retinal detachment
	Neovascular glaucoma
	Paediatric neovascular maculopathy
	Post-surgical macular edema
	Radiation retinopathy with macular edema
Alteplase	Subretinal macular hemorrhage
	Subretinal hemorrhage following age-related macular degeneration^1^
Amphotericin B	Corneal abscesses caused by *Candida*
Bevacizumab	Choroidal hemangioma
	Choroidal neovascularization associated with nevus, melanoma or other tumors
	Corneal neovascularization
	Cystic edema following brachytherapy
	Exudative retinal detachment after brachytherapy in choroidal melanoma
	Hemovitreous
	Macular edema associated with choroidal nevus, melanoma or other neoplasm
	Macular edema associated with macular telangiectasia
	Macular edema in von hippel lindau syndrome
	Neovascular glaucoma
	Ocular ischemic syndrome
	Post-surgical cystoid macular edema
	Radiation macular edema associated with choroidal melanoma
	Radiation retinopathy with macular edema
	Retinopathy of prematurity

1 Included in the list of Law 648/1996 since August 2025 (AIFA, 2025).

**TABLE 3:** Off-label drugs prescribed in specific ocular conditions (part II).

Drug	Pathology
Dexamethasone	Diabetic macular edema (early retreatment)
	Inflammatory macular edema
	Macular edema secondary to retinitis pigmentosa
	Macular edema secondary to vascular occlusion (early retreatment)
	Inflammation of the posterior segment caused by non-infectious uveitis (early retreatment)
	Radiation retinopathy with macular edema
Foscarnet	Panuveitis induced by varicella zoster virus
	Posterior herpetic uveitis
Ganciclovir	Severe posterior uveitis associated with cytomegalovirus retinitis
Mitomycin C	Trabeculectomy (antifibrotic)^1^
	Pterygium removal surgery (antifibrotic)
	Caruncular melanoma
	Caruncular squamous papilloma
Ranibizumab	Cystoid macular edema in irvine-gass syndrome
	Inflammatory macular edema
	Macular edema associated with macular telangiectasia
	Uveitic macular edema
	Neovascular glaucoma
	Juvenile idiopathic choroidal neovascularization
	Macular choroidal neovascularization due to Best’s disease
	Choroidal neovascularization secondary to angioid streaks
	Neovascularization due to coats’ disease
	Vasculitic retinal vascular occlusion
	Radiation retinopathy with macular edema
	Retinopathy of prematurity stage III^2^
	Vogt-koyanagi-harada syndrome
Rituximab	Ocular pemphigoid
Verteporfin	Central serous chorioretinopathy
Voriconazole	Candida chorioretinitis
	Candida endophthalmitis
	Candida vitreitis

1 Included in the list of Law 648/1996 since July 2016 (AIFA, 2016).

2 Approved by EMA, since 2019 (EMA, 2019).

## 4 Discussion

Off-label use of drugs in the field of ophthalmology is linked to the emerging of new therapies and the growing knowledge of multifactorial ocular diseases, as well as rare diseases. Indeed, off-label prescribing practice is often needed to handle complex ocular conditions, including eye surgeries or genetic diseases. Historically, off-label use of approved medications has been allowed by regulatory authorities, highlighting the importance of physicians’ responsibility to use or not to use off-label drugs based on risk/benefit ratio. Despite that, the real clinical off-label practice in the field of ophthalmology is poorly investigated.

### 4.1 Anti-VEGF drugs

In the recent past, the only noteworthy debate about off-label use in ophthalmology was represented by bevacizumab, an anti-VEGF drug approved for several different cancers, including metastatic colorectal and cervical cancers (Gerriets and Kasi, 2025). Particularly, bevacizumab is prescribed off-label to handle retinal diseases, as well as neovascular form of age-related macular degeneration (nAMD), macular edema, choroidal neovascularization (CNV), retinal vein occlusion (RVO), and proliferative diabetic retinopathy (PDR) (Gupta et al., 2010; Arevalo et al., 2011; Lushchyk et al., 2013; Stefanini et al., 2013; Vader et al., 2020). Due to its cost-effectiveness, bevacizumab is often employed by ophthalmologists to manage these retinal disorders. Particularly, the benefit of its intravitreal use in the treatment of neovascular retinal pathologies has been demonstrated in multiple clinical trials (Singh et al., 2024), making bevacizumab a good therapeutic option to prevent progressive blindness in these patients (van Asten et al., 2018). Indeed, it has been demonstrated that the ocular approved anti-VEGF drugs, as well as ranibizumab and aflibercept, despite their recognized efficacy, lead to overspending without additional health benefits in comparison to bevacizumab (Dakin et al., 2014; van Asten et al., 2018). The purpose of this study was to figure out off-label drugs prescribed in ophthalmology according to Law 94/1998 from January 2014 to December 2024 at the Department of Ophthalmology of Catania University Hospital. During these last 11 years, off-label drugs were prescribed to treat mainly adult patients with glaucoma or macular edema. 36.5% of the total prescriptions were performed in 2015 and 2016, closely correlated with the wide off-label use of mitomycin C, primarily in glaucoma trabeculectomy, until 2016. Mitomycin C is an antibiotic isolated from *Streptomyces caespitosus*, classified as an alkylating antineoplastic agent. Particularly, it is approved as systemic therapy of anal carcinoma, bladder carcinoma, breast carcinoma, head and neck malignancies, and some other gastrointestinal carcinomas (Sinawe and Casadesus, 2023). Additionally, mitomycin has been used off-label as topical adjunctive therapy in ocular surgery, including glaucoma filtering surgery, pterygium surgery, dacryocystorhinostomy, squint surgeries, corneal refractive surgeries, surgeries for ocular cicatrisation, as well as allergic conjunctivitis and ocular surface squamous neoplasia (Akpek et al., 2000; Abraham et al., 2006). Indeed, evidence in literature suggests an improvement of the surgical success rates of glaucoma filtering surgeries with mitomycin (Lama and Fechtner, 2003; Abraham et al., 2006; Wolters et al., 2021) and early clinical resolution of noninvasive ocular surface squamous neoplasia (Hirst, 2007). Particularly, its use in ophthalmic procedures is due to the inhibition of wound healing response, specifically through suppression of fibroblast and endothelial cells growth and replication, reducing fibrosis and scarring (Lama and Fechtner, 2003). Noteworthy, mitomycin represents 34.3% of the total prescriptions in this study, employed as antifibrotic to handle trabeculectomy and pterygium surgery. However, since July 2016, mitomycin was included in the list of medicinal products for human use that can be provided entirely by the NHS in Italy, according to Law 648/1996, as antifibrotic adjuvant in glaucoma trabeculectomy (AIFA, 2016), supported by a widely demonstrated increase in the success rate of glaucoma surgery (Abraham et al., 2006; Wolters et al., 2021; Nassiri et al., 2024). Additionally, several studies evidenced that adjunctive therapy with mitomycin as fibroblast proliferation inhibitor was effective in primary pterygium surgery, especially to reduce the high recurrence rates (Martins et al., 2016; Taher et al., 2022). In line with our results, several reports highlighted topical mitomycin use in ocular surface squamous neoplasia and conjunctival melanoma surgeries (Kurli and Finger, 2005; Nanji et al., 2013; Nassiri et al., 2024). After 2016 the number of off-label prescriptions drops to 1.7% in 2017 and 2018, according to mitomycin moving to the Law 648/96. Nevertheless, starting from 2019, there was an increasing trend in the number of off-label prescriptions, until 2024. According to our results, the main contributors to off-label prescriptions were anti-VEGF drugs. Particularly, among anti-VEGF agents, bevacizumab was the most prescribed drugs. Indeed, it has been employed outside its approved indications and administration route, to treat macular edema associated with choroidal melanoma or nevus, macular telangiectasia, or following radiotherapy. Accordingly, it has been demonstrated that bevacizumab intravitreal injection showed beneficial effects in improving macular edema associated with different eye conditions (Haritoglou et al., 2006; Mason et al., 2006b; Finger, 2008; Mackensen et al., 2008; Loukianou et al., 2010; Koay et al., 2011; Epstein et al., 2012; Gulyesil et al., 2023). Moreover, results of this study highlighted the off-label use of bevacizumab in two patients with choroidal hemangioma, a rare benign vascular tumor of choroid which can cause significant visual disturbance due to complications such as subretinal fluid, retinal detachment, retinoschisis and neovascular glaucoma (Shields et al., 2001; Lee et al., 2018). Noteworthy, some reports in literature showed that bevacizumab was effective to treat choroidal hemangioma, mainly as combined therapy (Hsu et al., 2011; Kwon et al., 2012; Durrani et al., 2022). In line with the well-known role of bevacizumab in the management of neovascular glaucoma (Chalam et al., 2008; Yazdani et al., 2009; Arcieri et al., 2015; Kwon and Sung, 2017; Al Sarireh et al., 2021; Hwang et al., 2025), this anti-VEGF agent was used off-label to treat 21 patients with neovascular glaucoma, a secondary form of glaucoma following diabetic retinopathy (DR) or retinal occlusion, usually associated with a poor visual prognosis. In this condition, bevacizumab demonstrated to be effective in reducing symptoms, iris neovascularization (NVI), angle neovascularization (NVA), and the need for surgical interventions, except in advanced and refractory cases (Mason et al., 2006a; Wakabayashi et al., 2008; Kotecha, 2011). Moreover, bevacizumab was employed off-label even to treat CNV associated with choroidal nevus, according to studies reporting that anti-VEGFs are effective in the treatment of CNV secondary to various macular and retinal disorders, including subretinal masses (Chiang et al., 2012; Cavalcante et al., 2014; Professional Association of German Ophthalmologists (Professional Association of German Ophthalmologists, BVA, et al., 2022). Additionally, as emerged from this study, bevacizumab was used off-label to treat ROP, a devastating neovascular disease of the retina caused by proliferation of abnormal fibrovascular tissue in premature infants (Dammann et al., 2023). Despite laser therapy is considered the gold standard treatment for ROP, the off-label use of anti-VEGF drugs has been reported since 2007. Noteworthy, bevacizumab was the first anti-VEGF treatment to be tested for ROP (Beccasio et al., 2022; Ortiz-Seller et al., 2024). Despite its advantages (Kusaka et al., 2008; Mintz-Hittner et al., 2011), concerns remain regarding adverse neurodevelopmental outcomes risk (Morin et al., 2016; Natarajan et al., 2019; Arima et al., 2020). However, a recent meta-analysis evidenced no increased risk of severe neurodevelopmental impairment after bevacizumab injection (Tsai et al., 2021). Although bevacizumab is not approved to eye diseases, it is widely prescribed by ophthalmologists due to its cost-effectiveness, safety and non-inferiority in comparison to other approved anti-VEGF agents for ocular use, and it is reimbursed in Italy according to Law 648/1996 (Elshout et al., 2018; Sangroongruangsri et al., 2018; van Asten et al., 2018). Among these latter, ranibizumab was the first anti-VEGF approved for ophthalmic use to treat wet AMD, diabetes or retinal occlusion-caused macular edema, proliferative PDR and myopic choroidal neovascularization (mCNV) (EMA, 2025). Additionally, based on results from the RAINBOW study, ranibizumab received approval for ROP in preterm infants from the European Medicines Agency (EMA) on September 2019 (Stahl et al., 2019; EMA, 2025). Indeed, as evidenced from this report, ranibizumab has been prescribed off-label to manage ROP, until 2019. Results from the present study showed that ranibizumab was prescribed off-label to manage cystoid macular edema (CME) related to Irvine-Gass syndrome, a condition that may develop after cataract surgery (Demirel et al., 2012; Fenicia et al., 2014). Additionally, ranibizumab was employed off-label to handle macular edema caused by inflammation, uveitis, macular telangiectasia, or following hadrontherapy for choroidal melanoma. These off-label uses were documented in literature; however, additional randomized controlled clinical trials need to be performed to elucidate the long-term clinical benefit (Acharya et al., 2009; Gonzalez, 2010; Ciarnella et al., 2012; Lim et al., 2018; Schefler et al., 2020). Moreover, as emerged from our report results, patients with neovascular glaucoma, idiopathic CNV, CNV secondary to angioid streaks, neovascularization due to Coats’ disease were treated with ranibizumab, in line with several studies (Mimoun et al., 2010; Lüke et al., 2013; Tilleul et al., 2016; Katsanos et al., 2018; Lai et al., 2018; Gaillard et al., 2014; Yang et al., 2016; Li et al., 2017; Zhang et al., 2018; Nowara et al., 2021). Coats’ disease is an idiopathic, rare ocular disorder affecting young males characterized by retinal telangiectasis, exudation, and retinal detachment. The use of intravitreal anti-VEGFs demonstrated to be effective and safe in these patients, nevertheless, additional longitudinal studies with longer follow up in a larger cohort of patients affected by Coats’ disease are needed (Cennamo et al., 2021; Zhou et al., 2024). One pediatric patient has been treated with ranibizumab for CNV due to Best’s disease or Best vitelliform macular dystrophy, a rare genetic disorder due to the mutation of *BEST1* (or *VMD2*, *TU15B*) gene, typically presenting in childhood and complicated by CNV (Spaide et al., 2006; Johnson et al., 2017); some literature reports suggested a favorable prognosis after anti-VEGFs intravitreal injection in patient with macular or choroidal neovascularization (Heidary et al., 2011; Fayed, 2022), though further studies supporting this off-label use are needed. Similarly, aflibercept was employed outside its approved indications to manage CME associated Irvine-Gass syndrome, neovascular glaucoma and macular edema secondary to various disorders, in line with some literature reports (Lin and Tsai, 2018; Fallico et al., 2019; Murray et al., 2019; Akay et al., 2020). Additionally, aflibercept was even used off-label to handle macular edema associated with inflammation. Indeed, the anti-inflammatory effect of aflibercept is well-documented (Kotake et al., 2018; Lazzara et al., 2019; Li et al., 2024). Lastly, according to our results, pediatric neovascular maculopathy was even treated off-label with aflibercept, with some evidence in literature regarding its intravitreal use in idiopathic pediatric CNV (Barth et al., 2016; Viana et al., 2023). However, larger studies are required to assess the long-term efficacy and safety profile of aflibercept in pediatric patients.

### 4.2 Anti-inflammatory drugs

Biodegradable dexamethasone intravitreal implant is approved to treat macular edema following branch retinal vein occlusion (BRVO) or central retinal vein occlusion (CRVO), non-infectious uveitis affecting the posterior segment of the eye and diabetic macular edema in patients who are pseudophakic or are phakic and scheduled for cataract surgery. Results of this study highlighted the early retreatment with dexamethasone intravitreal implant outside the approved posology and method of administration to handle macular edema following vascular occlusion, inflammation of the posterior segment caused by non-infectious uveitis and diabetic macular edema. Indeed, according to the SmPC, retreatment may be performed after approximately every 6 months due to very limited information on shorter dosing intervals (EMA, 2025b). Several controlled clinical trials have shown the efficacy and safety of intravitreal dexamethasone treatment with an interval <6 months (Bucolo et al., 2018; Yoon et al., 2018). The optimal retreatment schedule for patients requiring multiple injections should be further explored. Finally, dexamethasone was employed off-label to manage inflammatory macular edema, radiation macular edema associated with choroidal melanoma, and macular edema caused by retinitis pigmentosa, in line with some literature evidence (Baillif et al., 2013; Ahn et al., 2014; Tomkins-Netzer et al., 2014; Caminal et al., 2015; Park et al., 2020).

### 4.3 Other drugs

5-fluorouracil is a chemotherapy drugs used to treat various neoplasms, e.g., gastric, pancreatic, breast, and colorectal cancer, together with some dermatologic conditions. It was prescribed off-label as topic treatment to manage conjunctival intraepithelial neoplasia and squamous cell carcinoma of the conjunctiva/cornea, including recurrent forms. Noteworthy, several studies reported 5-fluorouracil as effective and non-invasive method to manage these conditions (Midena et al., 2000; Al-Barrag et al., 2010; Bakal et al., 2024). Another drug prescribed off-label by ophthalmologists was alteplase, which is a thrombolytic agent approved to handle acute ischemic stroke, pulmonary embolism, acute myocardial infarction, and occluded catheters (Reed et al., 2025). Results of this report showed alteplase off-label use to treat subretinal macular hemorrhage, in particular following AMD. Indeed, it has been showed that combined therapy with recombinant tissue plasminogen activator (tPA) and anti-VEGFs is effective in managing submacular hemorrhage associated with wet-AMD (Murphy et al., 2024; Veritti et al., 2024). It is noteworthy that since August 2025 this drug can be prescribed and reimbursed in Italy according to Law 648/1996 for subretinal macular hemorrhage in patients with AMD. Another relatively common cause of central vision loss is central serous chorioretinopathy (CSC). It is a retinal disorder characterized by localized detachment of the macula, secondary to subretinal fluid leakage, mostly seen in young men (Liu et al., 2016). Usually, it resolves spontaneously within 3–4 months, but some cases require treatment to avoid permanent loss of vision (van Rijssen et al., 2019). Verteporfin photodynamic therapy (PDT) has been recognized as the treatment of choice for CSC (Zhao et al., 2015; van Dijk et al., 2018; Kim et al., 2025). Results from this study exhibited off-label prescriptions of verteporfin to manage this complex ocular condition. In the contest of eye complications associated with vision loss and long-term morbidity, *candida* endophthalmitis (CE) and chorioretinitis represent a challenge to ophthalmologists, although uncommon. Indeed, diagnosis, as well as therapeutic options are characterized by restricted evidence and discrepancies in the clinical practice (Permpalung et al., 2025). Ocular fungal infections can be treated by systemic or intravitreal injection of active agents. Voriconazole is a triazole antifungal medication approved for systemic use to treat *Candida* and *Aspergillus* infections (Patel and Zito, 2025). As emerged from the present study, voriconazole has been prescribed off-label by specialists to manage *Candida* chorioretinitis, endophthalmitis and vitreitis, based on clinical data on its use by intravitreal injections (Sen et al., 2006; Vasconcelos-Santos and Nehemy, 2009; Silva et al., 2015; Bienvenu et al., 2020; Sim et al., 2020; Abu Talib et al., 2021; Patel et al., 2021; Gentile et al., 2025). Noteworthy, the herein reported off-label use of voriconazole is related not only to its approved indications, but even to the administration route. The antifungal amphotericin B, approved to treat systemic fungal infections, was employed topically off-label to manage corneal abscesses caused by *Candida,* supported by several studies which describe the use of the drug to treat severe fungal keratitis resistant to conventional therapy (Noor and Preuss, 2024, Garcia-Valenzuela and Song, 2005; Hu et al., 2016; Nada et al., 2017; Aydin et al., 2020; Zemba et al., 2023). Foscarnet is an antiviral agent used to treat cytomegalovirus (CMV) and CMV-associated ophthalmic retinitis in individuals diagnosed with AIDS and who are unable to tolerate ganciclovir, as well as for patients with drug-resistant CMV who have failed ganciclovir therapy. Additionally, this antiviral drug is a treatment option in immunocompromised patients with mucocutaneous herpes simplex virus (HSV) who exhibit resistance to acyclovir (Garikapati and Nguyen, 2025). As highlighted from results of this study, foscarnet was employed off-label to handle panuveitis induced by varicella zoster virus (VZV) and posterior herpetic uveitis. Indeed, systemic antivirals + intravitreal antiviral therapy are recommended as first-line option for severe ophthalmic panuveitis caused by herpes viruses, combining high-dose valaciclovir orally or intravenous acyclovir with intravitreal foscarnet (Debiec et al., 2021; Kalogeropoulos et al., 2024).

Ganciclovir is an antiviral drug approved to treat herpes virus infections, as well as CMV-induced retinitis in immunocompromised adult patients (Gupta and Shorman, 2025). The present study showed the off-label use of intravitreal injections of ganciclovir to treat an immunocompromised patient affected by severe bilateral posterior uveitis associated with CMV retinitis, with poor response to systemic therapy. The standard treatment of severe CMV infections of the eye consists of systemic antivirals, in particular intravenous ganciclovir (Tasiopoulou et al., 2023). Furthermore, a body of evidence supports the role of intravitreal antiviral drugs, including ganciclovir, in immunocompromised patients with CMV retinitis (Miao et al., 2013; Singh et al., 2013; Agarwal et al., 2014; Jeon and Lee, 2015; Qian et al., 2018; Shapira et al., 2018). Further research is needed to improve visual outcome of these clinical conditions. Finally, one patient with ocular involvement of pemphigoid disease was treated with rituximab, an anti-CD20 monoclonal antibody approved to manage different conditions, as well as various lymphoproliferative and autoimmune disorders (Hanif and Anwer, 2025). Pemphigus vulgaris (PV) is a rare autoimmune disease leading to cutaneous and mucosal surfaces blistering, with occasional involvement of ocular surface (Memar et al., 2020; Ingold et al., 2025). Conventional treatment includes high-dose corticosteroids, immunosuppressive drugs, and intravenous immunoglobulin. Several reports showed the efficacy of rituximab in patients with refractory PV, but also in those with ocular pemphigoid (Ahmed et al., 2006; Gürcan et al., 2009; Foster et al., 2010; Feldman and Ahmed, 2011). As evidenced from these results, the off-label use in ophthalmology is growing, particularly due to the necessity to ameliorate the patient care. Noteworthy, considering that this was a retrospective analysis of data collected from one single center, results cannot be generalized, representing a limitation of this study. In addition, this study did not provide information regarding patient’s treatment outcomes.

In conclusion, personalized medicine, and gene therapy firmly contribute to the evolving of off-label drugs use. Additionally, off-label practice constitutes an important starting point for future research to repurpose drugs for new indications, providing innovation to the clinical practice (Rusz et al., 2021). Obviously, related ethical, legal, and clinical challenges need to be addressed. In particular, robust evidence should be collected to support the prescription of some off-label drugs; therefore, high-quality extensive observational studies and randomized controlled trials are still crucially needed. Noteworthy, guidelines and standards for drugs off-label use are necessary in order to manage concerns about drugs safety and efficacy in order to ensure patient’ well-beings.

## Data Availability

The raw data supporting the conclusions of this article will be made available by the authors, without undue reservation.
